# Dual Role of Inflammasome Adaptor ASC in Cancer

**DOI:** 10.3389/fcell.2020.00040

**Published:** 2020-02-04

**Authors:** Maria Pia Protti, Lucia De Monte

**Affiliations:** ^1^Tumor Immunology Unit, Istituto di Ricovero e Cura a Carattere Scientifico (IRCCS) San Raffaele Scientific Institute, Milan, Italy; ^2^Division of Immunology, Transplantation and Infectious Diseases, Istituto di Ricovero e Cura a Carattere Scientifico (IRCCS) San Raffaele Scientific Institute, Milan, Italy

**Keywords:** ASC/TMS1, tumor cells, myeloid cells, tumor suppressor gene, inflammasome, IL1, IL-18

## Abstract

Apoptosis-associated Speck-like protein containing a CARD (caspase activation and recruitment domain) (ASC), also called PYCARD/Target of Methylation-induced Silencing-1 (TMS1), was originally discovered as a protein that forms aggregates (“specks”) in human leukemia cells treated with chemotherapeutic agents. Its expression was found to be silenced by methylation in many human tumors, preventing tumor cells from undergoing apoptosis and supporting its role as a tumor suppressor. Subsequently, ASC was also identified as a central adaptor molecule of the inflammasome complex, which mediates the secretion of inflammatory cytokines (i.e., IL-1β and IL-18). Inflammatory cytokines have been shown to mediate tumor-promoting functions. Thus, in the context of cancer development and progression, ASC may exert opposing functions, i.e., be either tumor-suppressing by inducing tumor cell apoptosis, or tumor-promoting by favoring secretion of inflammatory cytokines (by tumor cells and/or tumor infiltrating myeloid cells) within the tumor microenvironment. Here, we report and discuss this dual role of ASC by also considering the final contribution of each of its two main functions in several cancer types, taking into consideration the correlation between ASC expression, clinical correlates, and patients’ survival. ASC and inflammasome targeting strategies are being developed. However, before the use of such treatments in clinical practice, it is fundamental to better dissect the role of ASC in different tumors, in order to privilege or avoid their use in those tumors in which ASC exerts an anti-tumor or pro-tumor function, respectively.

## Introduction

Apoptosis-associated speck-like protein containing a CARD, i.e., caspase activation and recruitment domain (ASC), also called PYCARD or TMS1 (Target of Methylation-induced Silencing-1), was originally identified as a cytosolic protein, forming large aggregates called specks in HL-60 cells after induction of apoptosis by retinoid acid and other anti-tumor drugs (Masumoto et al., 1999). TMS1 was independently identified during a screening for targets of methylation-associated gene silencing in human breast cancer cells (Conway et al., 2000). Structurally, ASC/TMS1 is a 22 kDa protein containing a N-terminal pyrin (PYD) domain and a C-terminal CARD domain (Masumoto et al., 1999; Martinon et al., 2001, 2002). ASC/TMS1 is expressed in several normal epithelial and immune cells, where it localizes in the nucleus and, upon activation, redistributes in the cytoplasm and eventually aggregates in specks (Masumoto et al., 1999; McConnell and Vertino, 2000).

ASC was shown to be downregulated to various extents in several human cancers when compared to the normal tissue counterpart or non-tumor adjacent tissue, suggesting a role as a tumor-suppressor (Table 1). This function was supported by experiments, in which ectopic ASC expression induced tumor cells to apoptosis (Conway et al., 2000; Ohtsuka et al., 2004, 2006; Parsons and Vertino, 2006; Ramachandran et al., 2010; Hong et al., 2013), whereas knocking down endogenous ASC inhibited tumor cell death (Ohtsuka et al., 2004; Parsons et al., 2009; Hong et al., 2013). ASC was then identified as an inflammasome adaptor molecule for caspase-1 activation, pro-interleukin (IL)-1β, and pro-IL-18 cleavage and maturation (Martinon et al., 2002; Srinivasula et al., 2002), supporting a pro-inflammatory function. By promoting inflammation and specifically IL-1 release, ASC may indirectly exert pro-tumor activities by inducing chronic inflammation, angiogenesis, activation of the IL-17 pathway, myeloid-derived suppressor cell differentiation, and macrophage recruitment, invasion, and metastasis (Mantovani et al., 2018).

**TABLE 1:** ASC/TSM1 up- and down-regulation (methylation status) and clinical correlates in human cancers.

Tumor type	Expression/methylation status in normal tissue	Expression/methylation status in cancer tissue	Clinical correlates	Clinical outcome	References
Breast cancer	Unmethylated in normal breast tissue (*n* = 3)	Methylation in 40% (11 of 27) of tumors and 44.4% (8 of 18) of paired adjacent tissues	NE	NE	Conway et al., 2000
SCLC, NSCL, breast cancer	Unmethylated in normal lung tissue (*n* = 18) and 7% (2 of 30) of normal breast tissues	Methylation in 41% (13 of 32) of SCLC, 40% (28 of 70) of NCSLC and 32% (20 of 63) of breast cancer	None	NE	Virmani et al., 2003
NSCLC	Methylation in 12.9% (9 of 70) of normal lung tissues	Methylation in 47.1% (33 of 70) of lung tumors	Methylation as an independent unfavorable prognostic factor	Patients with unmethylated tumors had better survival	Zhang et al., 2006
Lung cancer	Expressed in normal tissue (*n* = 6) and pre-cancerous lesions (*n* = 10) Methylation in 1 of 23 pre-cancerous lesions	Reduced expression in 75% (30 of 40) of tumors Methylation in 27% (41 of 152) of tumors	Methylation correlates with lymphatic invasion, lymph node metastases and advanced stage	Methylation in sputum DNA predicts prognosis in patients resected for early stage disease	Machida et al., 2006
Colorectal cancer	Methylation in 12.5% (2 of 16) normal tissue	Methylation in 25% (4 of 16) of tumors	Methylation associates with lack of nodal metastases	NE	Ohtsuka et al., 2006
Colorectal cancer	Methylation in 20% (2 of 10) of adjacent normal tissues	Partial methylation in 60% (6 of 10) of tumors	NE	NE	Yokoyama et al., 2003
Colorectal cancer	Unmethylated in normal tissue (*n* = 11) and adenomas (*n* = 30)	Methylation in 17% (20 of 115) of tumors	Methylation more common in right-sided tumors and in late stages	NE	Riojas et al., 2007
Melanoma	Highly expressed in melanocytic nevi (*n* = 18)	Absent or reduced in 62.5% (20 of 32) of melanomas	NE	NE	Guan et al., 2003
Melanoma	Highly expressed in normal melanocytes	Downregulated in primary (*n* = 6) and in metastatic lesions (*n* = 6)	NE	NE	Liu et al., 2013
Ovarian cancer	Unmethylated in normal ovary tissue (*n* = 4)	Methylation in 19% (15 of 80) of tumors	Methylation correlates with clear cell-type tumors	No correlation with prognosis	Terasawa et al., 2004
Ovarian cancer	NE	Methylation in 40% (8 of 20) of tumors	NE	NE	Akahira et al., 2004
Glioblastoma	Expressed and unmethylated in normal brain (astrocytes)	Methylation in 43% (10 of 23) of tumors	None	Increased survival in patients with unmethylated tumors	Stone et al., 2004
Glioblastoma	NE	Methylation in 21.05% (12 of 57) of tumors	Methylation increased (4 of 7) in long-term survivors	Increased survival in patients with methylated tumors	Martinez et al., 2007
Neuroblastoma	NE	Methylation in 31% (45 of 145) of tumors	Methylation correlates with advanced disease	Methylation associates with reduced survival	Alaminos et al., 2004
Medulloblastoma	NE	Expression in 60% (9 of 11) of tumor samples	NE	NE	Knight et al., 2015
Prostate cancer	Unmethylated in normal tissues (*n* = 14) Methylation in 28% (11 of 40) of adjacent tissue	Methylation in 65% (38 of 58) of tumors and 64% (7 of 11) of high-grade intraepithelial neoplasia	Methylation in adjacent tissue correlates with biochemical recurrence	None	Collard et al., 2006
Prostate cancer	NE	Methylation in 63.6% (42 of 66) of tumors and 35% (12 of 34) of prostate hyperplasia	Methylation more prevalent in the white race	NE	Das et al., 2006
Gastric cancer	NE	Methylation in 32.1% (20 of 80) of tumors	None	Reduced survival for patients with methylation	Kato et al., 2008
Gastric cancer	Expression higher than in tumor tissue and unmethylated (*n* = 40)	Methylation in 34% (68 of 200) of tumors	Methylation correlates with tumor size and lymph node metastases	Reduced survival for patients with methylation	Wu L. et al., 2016
Gastric cancer	Expression lower than in tumors in two patient cohorts (*n* = 10 and *n* = 18)	Expression higher than in non-tumor adjacent tissues in the same patient cohorts	Correlation between ASC and IL18 mRNA levels	NE	Deswaerte et al., 2018
Cervical cancer	Methylation in 2.5% (2 of 80) of non-tumor adjacent tissue	Methylation in 6.2% (5 of 80) of tumors	None	NE	Kordi Tamandani et al., 2009
Renal cell carcinoma	Highly expressed in adjacent non-cancerous tissues (*n* = 67) Methylation in 12% of normal tissue	Downregulation in tumor compared to normal tissues (*n* = 67) Methylation in 41.1% (83 of 202) of tumors	Methylation correlates with higher nuclear grade	NE	Liu et al., 2015
Oral squamous cell carcinoma	Highly expressed in normal oral mucosa (*n* = 6)	Downregulation in tumors as a function of differentiation grade	Correlation with clinical features	Better survival in patients with higher ASC score (% of positive cells)	Shimane et al., 2013
Oral squamous cell carcinoma	Downregulated in normal tissue compared to tumors (*n* = 20)	Upregulated in tumors compared to normal mucosa (*n* = 111)	ASC increased expression as an independent predictor of survival	ASC expression correlated with reduced survival	Wu C.S. et al., 2016
Cutaneous squamous cell carcinoma	Expressed in normal tissue	Loss of expression in de-differentiated G3 tumors	None	NE	Meier et al., 2016
Pancreatic ductal adeno-carcinoma	Weak staining in adjacent non-tumor tissue (*n* = 41)	ASC expression in all tumor samples; i.e., 90% (37 of 41) of tumor cells and 100% of TAMs (*n* = 41)	NE	Increased survival in patients with ASC mRNA expression inferior to the median values	Brunetto et al., 2019

*NE, not evaluated.*

In this review, we summarize and discuss data from the literature describing both ASC functions (pro-apoptotic and pro-inflammatory), their implication in anti-tumor or pro-tumor activity, the correlation between ASC expression/upregulation or down-regulation by aberrant methylation in tumor and clinical correlates, and survival in neoplastic patients.

## ASC/TMS1 as Tumor Suppressor

**FIGURE 1:**
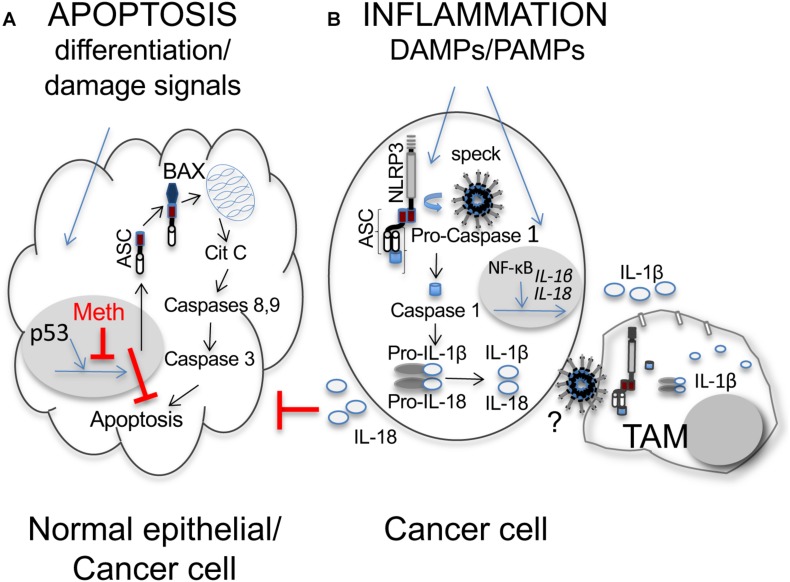
Dual role of ASC/TMS1 in cancer. **(A)** Pro-apoptotic function. In normal epithelial cells differentiation and stress/damage cell signals induce the onco-suppressor p53 to activate transcription of several genes, among these ASC. Activated ASC binds to Bax and the ASC-Bax complex translocates to mitochondria mediating Citochrome C (Cit C) release. Cit C activates the initiator caspases-8 or -9, which in turn activate caspase-3 and the apoptotic cascade. At difference with normal epithelial cells, in cancer cell methylation (Meth) of the promoter region of ASC induces gene silencing and inhibits apoptosis, thus contributing to cell survival and tumor development (Hanahan and Weinberg, 2011). **(B)** Pro-inflammatory function in cancer cells. In cancer cells and myeloid cells (such as TAMs), recognition of pathogen- or damage-associated molecular patterns (PAMPs, DAMPs) induces the assembly/polymerization of the inflammasome molecular complex, which in cancer cells is otherwise often constitutively activated as a result of genetic lesions (Kolb et al., 2014; Kantono and Guo, 2017; Karan, 2018; Karki and Kanneganti, 2019). The inflammasome is composed by a multimerized module formed by a sensor Nod Leucine-Rich Repeat-containing receptor, such as NLRP3, bound to the pyrin domain of the adaptor ASC, which in turn is bound, through its CARD domain, to pro-caspase-1. The multimerized complex forms a speck that induces caspase-1 activation. Caspase-1 can then catalyze the proteolytic cleavage and activation of IL-1β and IL-18. Sensing of PAMPS or DAMPS also induces activation of NF-κB, which translocates to the nucleus and activates transcription of pro-IL-1β and pro-IL-18. ASC specks and inflammatory cytokines (IL-1β and IL-18) are released from cancer cells, through mechanisms not completely elucidated (?), and captured by TAMs contributing to massive IL-1β release with auto-activation of cancer cells, as well as triggering of other immune or stromal cell components in the tumor microenvironment. IL-18 can contribute to cancer cell proliferation by inhibiting caspase-8 mediated apoptosis.

Apoptosis is a regulated cell death process, which results in the clearance of dying cells with minimal damage to surrounding tissues (Galluzzi et al., 2018; Singh et al., 2019). Once cell damage is detected, a series of cysteine-aspartic proteases called caspases are activated. These include initiator caspases (caspase-8 and -9), which in turn activate executioner caspases (e.g., caspase-3), initiating a cascade of events resulting in DNA fragmentation, destruction of nuclear proteins and cytoskeleton with chromatin and cytoskeleton condensation, expression of ligands for phagocytic cells, and the formation of apoptotic bodies (Figure 1A). Apoptotic bodies are removed by macrophages before their fragmentation, reducing the risk of collateral damage to adjacent cells.

Apoptosis is initiated by intracellular or extracellular microenvironmental perturbations that trigger the intrinsic (mitochondrial outer membrane permeabilization-mediated) or the extrinsic (death receptor-mediated) pathways, respectively. Failure of apoptosis and consequent accumulation of damaged cells is associated with tumor formation (Galluzzi et al., 2018; Singh et al., 2019). Indeed, tumor cells develop strategies to limit or circumvent apoptosis, such losing the p53 tumor suppressor function, increasing expression of anti-apoptotic regulators (the Bcl-2 protein family) or survival signals (Igf1/2), downregulating pro-apoptotic factors (Bax), or avoiding the extrinsic ligand-induced death pathway (Hanahan and Weinberg, 2011).

As reported above, ASC was found to be silenced by DNA methylation in cancer cells from different tumors, indicating a role as an anti-tumor pro-apoptotic factor (Table 1).

A mechanism of the caspase-9-dependent pro-apoptotic function for ASC/TMS1 was first described in McConnell and Vertino (2000), where apoptosis was accompanied by the redistribution of ASC/TMS1 from the cytoplasm to perinuclear spherical structures similar to the large aggregates (i.e., specks) described in Masumoto et al. (1999). Pro-apoptotic activity as well as spherical structure formation were dependent on the CARD portion of molecule. Whereas the apoptotic activity of ASC was blocked by caspase inhibition, the formation of the spherical structures was not, suggesting that ASC redistribution precedes caspase activation.

Levine et al. (2003) investigated at the molecular level the regulation of TMS1 silencing by methylation in human breast cancer cells. The authors characterized the ASC/TMS1 locus with regard to the methylation status by comparing the fine mapping of methylation in normal mammary epithelial cells, breast cancer cell lines (either positive or negative for TMS1 expression), and primary breast tumors. The region surrounding the transcription start site was found to be crucial for TMS1 expression. In primary tumors, the methylation-associated silencing of TMS1 was usually present in a subset of tumor cells, while TMS1 negative cell lines showed a nearly complete methylation at each CpG islands in all alleles. The authors suggested a model, in which dense methylation and gene silencing are events tightly combined and involving local remodeling of CpG island chromatin (Levine et al., 2003). A similar association between ASC silencing and methylation status was reported in several other neoplastic diseases (Table 1).

The mechanisms of ASC-mediated apoptosis were studied using *in vitro* cell line models (Ohtsuka et al., 2004). ASC was shown to induce apoptosis in a p53-dependent manner. In response to genotoxic agents, p53 induced ASC expression by binding to its promoter and activating its transcription. In this model, Bax, a pro-apoptotic protein that causes p53-mediated mitochondrial dysfunction, interacted with ASC through its Pyrin domain in the cytoplasm. The Bax-ASC complex then translocated to the mitochondria and induced the release of cytochrome C, which in turn triggered apoptosis through the activation of caspase-9, -2, and -3. Collectively, this study demonstrated that ASC acts as an adaptor molecule for Bax, and regulates a p53-Bax mitochondrial pathway of apoptosis.

The effects of ASC expression on p53-mediated chemosensitivity were subsequently evaluated in colon cancer (Ohtsuka et al., 2006). ASC overexpression in p53-expressing tumor cells promoted cell death and increased chemosensitivity, suggesting that methylation-induced silencing of ASC might cause resistance to p53 mediated chemosensitivity, and that restoration of ASC expression should increase chemotherapy efficacy.

A mechanism of resistance to anoikis mediated by TMS1/ASC has been reported in breast cancer early carcinogenesis (Parsons et al., 2009). Anoikis is a form of programed cell death provoked in epithelial cells by detachment from the extracellular matrix. Resistance to anoikis is acquired by epithelial cells during carcinogenesis. TMS1/ASC expression was reduced in a subset of *in situ* lesions where the epithelial cells had filled the breast duct, and in 16% of the invasive ductal carcinomas. These data are suggestive of a possible role of TMS1/ASC in the transition from *in situ* to invasive lesions. *In vitro* forcing suspension of breast epithelial cells resulted in TMS1/ASC expression, which preceded that of the proapoptotic protein Bim (known to be upregulated during anoikis). TMS1/ASC knockdown inhibited Bim induction, procaspase-8 cleavage, and led to persistence of MAPK/ERK survival pathways, suggesting that TMS1/ASC silencing contributes to the resistance to anoikis.

One study (Liu et al., 2015) evaluated epigenetic alteration and the biological function of ASC/TMS1 in renal cell carcinoma. ASC/TMS1 was downregulated in tumor cell lines and tumor compared to normal tissue samples. Downregulation of ASC/TMS1 correlated with its promoter methylation and could be restored by treatment with demethylating agents. ASC/TMS1 re-expression inhibited tumor cells viability and colony formation, arrested cell cycle, induced apoptosis, suppressed cell invasion and repressed tumorigenicity in immunodeficient mice. All these functions were partially regulated by activation of the p53 signaling.

More recently, the effects of ASC on cell viability were studied using different cell density conditions (Kitazawa et al., 2017). The authors found that at high-density, cell viability was suppressed by ASC-dependent apoptosis induced by cleavage of caspase-9, and by suppression of the NF-κB related X-linked inhibitor-of-apoptosis protein expression. Caspase-9 cleavage was partially dependent on enhanced gap junction formation.

An anti-tumor but not pro-apoptotic mechanism for ASC, which involves caspase-8, was identified in Okada et al. (2016). The authors found that ASC ablation in murine tumor cell lines *in vitro* enhanced cellular motility and invadopodia formation through cytoskeletal reorganization, as well as Src accessibility to caspase-8 for ensuing phosphorylation (*p*-caspase-8 is pro-metastatic) and cellular migration. Indeed, *in vivo* this effect was associated to reduced metastatic potential.

In summary, a role for ASC/TMS1 as a pro-apoptotic tumor suppressor factor has been assessed in different tumors where it was found to be downregulated in variable percentages in primary tumors. Mechanistic studies clarified that lack of ASC/TMS1 protein expression was associated at the molecular level with hypermethylation of its promoter region. *In vitro* ASC/TMS1 expression could be at least partially restored upon treatment with demethylating agents, and forced expression of the ASC/TIMS1 gene by transfection in negative tumor cell lines endowed them with apoptotic capability. Future studies are needed to determine what stimulate ASC to induce apoptosis in cancer, as upregulation of ASC *per se* is possibly not sufficient.

## ASC as Inflammasome Adaptor Molecule

Inflammation, which is an enabling hallmark of cancer (Hanahan and Weinberg, 2011), contributes to tumor development/progression through several mechanisms. For example, through providing soluble molecules to the tumor microenvironment (including growth, survival, and proangiogenic factors); through enzymes, which modify the extracellular matrix to favor angiogenesis, invasion and metastasis; and through signals to activate epithelial to mesenchymal transition (Mantovani et al., 2008; Hanahan and Weinberg, 2011).

Inflammatory responses originate in response to microbial and danger signals, through the activation of large cytoplasmic protein complexes, termed inflammasomes, which are essential for the production of active IL-1β and IL-18 cytokines (Martinon et al., 2002). ASC plays a central role during inflammasome activation by interacting via its PYD domain with pattern recognition receptors (e.g., NLRP3), and via its CARD domain with pro-caspase-1, leading to caspase-1-activation and proIL-1β and proIL-18 maturation (Martinon et al., 2002; Srinivasula et al., 2002).

The role of inflammasomes in cancer has been recently discussed elsewhere (Kolb et al., 2014; Kantono and Guo, 2017; Karan, 2018; Karki and Kanneganti, 2019; Van Gorp and Lamkanfi, 2019). We refer readers interested in comprehensive summaries of the field to those reviews.

We focus here on ASC for which a role in tumor development/progression, as an inflammasome adaptor molecule, occurring through different indirect mechanisms, has been reported (Figure 1B).

In a mouse model of epithelial skin carcinogenesis, the function of ASC in tumor initiation or suppression was studied using conditional ASC knockout mice (Drexler et al., 2012). IL-1 receptor- or caspase-1-deficient mice showed reduced cancer incidence and tumor numbers compared to wild-type controls. However, ASC-deficient mice were not protected. To differentiate putative tumor-suppressive from tumor-promoting functions, mice deficient in ASC in keratinocytes or in myeloid cells were generated. While mice deficient in ASC in keratinocytes developed more tumors than controls, those deficient in myeloid cells were protected. ASC-deficient keratinocytes enhanced their proliferation both *in vitro* and *in vivo*, possibly through p53 activation. ASC deletion in myeloid cells was associated with significantly reduced IL-1β in the tumor. According to the dual role of ASC in skin carcinogenesis, ASC protein expression was lost in human cutaneous squamous cell carcinoma, but not in inflammation-induced epidermal hyperplasia, such as psoriatic skin lesions (Drexler et al., 2012; Meier et al., 2016). Collectively, these studies suggested that ASC in different tumor components (i.e., epithelium vs stroma) may influence tumor growth in opposite directions.

The role of ASC in melanogenesis was studied using primary and metastatic melanoma cell lines (Liu et al., 2013). The results showed that ASC has tumor stage-dependent dual roles in tumorigenesis (i.e., suppresses tumor growth in primary melanoma while it promotes tumor growth of metastatic melanoma) by different regulations of NF-κB activity. In primary melanoma ASC inhibited phosphorylation of IκB Kinase and decreased NF-κB activity with low pro-IL-1β synthesis and release. In metastatic melanoma, auto-active IL-1 receptor and other signaling pathways resulted in reduced NF-κB inhibition by ASC in the presence of sustained auto-active inflammasome, leading to spontaneous IL-1β synthesis and release (Liu et al., 2013). These data further clarify the mechanisms of previous results, in which constitutively assembled and activated NLRP3 inflammasome resulted in spontaneous IL-1β secretion in metastatic melanoma (Okamoto et al., 2010). Interestingly, ASC was also reported to differentially regulate NF-κB activity toward either enhancement or inhibition, depending on the ratio of its levels to other ASC-binding proteins (e.g., Cryopyrin/PYPAF-1, Pyrin, PYPAF-7, and caspase-8 for enhancement, and IKK complex components IKKα, IKKβ and IKKγ for inhibition, respectively) (Manji et al., 2002; Stehlik et al., 2002; Hasegawa et al., 2005).

A pro-tumorigenic function for ASC, through its effector cytokine IL-18, was recently described in gastric cancer (Deswaerte et al., 2018), where ASC was significantly upregulated in the tumor compared to normal gastric tissue. Mechanistic studies were performed on spontaneous mouse models of gastric cancer. In these mice, ablation of ASC was associated with reduced NF-κB, caspase-1 activation, and enhanced numbers of caspase-3 and -8 positive tumor cells, suggesting a tumor-promoting function for ASC by limiting apoptosis in the gastric epithelium, independently of its pro-inflammatory effects. Similarly, ASC deletion or IL-18 blockade in tumor cells increased apoptosis (Figure 1), and in tumor samples elevated levels of IL-18 and ASC mRNA showed positive correlation, supporting a role for IL-18 in protecting tumor cells from apoptosis. Collectively, the study suggests the existence of a novel ASC/IL-18/NF-κB signaling axis that augments tumor cell survival in gastric cancer.

A novel role for extracellular ASC released by tumor cells in pancreatic cancer was recently reported (Brunetto et al., 2019). In this study, IL-1α and IL-1β from tumor cells and tumor associated macrophages (TAMs) were key to inducing cancer-associated fibroblasts (CAFs) to secrete the thymic stromal lymphopoietin (TSLP), which in turn drives predominant tumor-promoting Th2 cells, through the activation of resident dendritic cells with Th2 polarizing capability (De Monte et al., 2011; Protti and De Monte, 2012). It has been shown that ASC assemble and form specks that are released by dying cells, and phagocytosis of ASC specks by macrophages leads to IL-1 maturation through caspase-1 activation (Baroja-Mazo et al., 2014; Franklin et al., 2014), suggesting that extracellular ASC specks serve as danger signals (Figure 1B). Indeed, *in vitro* studies showed that ASC released by tumor cells induced IL-1β release by macrophages, and CAFs, activated with the supernatant of ASC positive tumor cell-conditioned macrophages, secreted TSLP. Collectively, ASC in pancreatic cancer exert an indirect role in driving predominant Th2-type inflammation, through a complex cross-talk between tumor cells and its microenvironment. Importantly, in a murine model, in which ASC-deficient mice were orthotopically implanted with KPC-derived tumor cells [i.e., tumor cells derived from a spontaneous mouse model of pancreatic cancer (Hingorani et al., 2005)], tumors grew at lower rate in ASC-deleted compared to wild-type mice (Daley et al., 2017). Collectively, these studies indicate a tumor-promoting function for ASC in pancreatic cancer.

## ASC Expression/Aberrant Methylation and Clinical Outcome

The levels of ASC mRNA and protein expression have been investigated in several neoplastic diseases using reverse transcription polymerase chain reaction. This was in order to specifically address the presence of a hypermethylated status, and, by immunohistochemistry, to morphologically distinguish ASC expression in tumor cells and myeloid cells within the tumor microenvironment. In the majority of studies, the levels of ASC expression in the normal tissue counterpart and/or in non-tumor adjacent tissues were evaluated for comparison (Table 1).

In this section, we report and discuss those studies, in which the relevance of ASC expression and/or hypermethylation in relation to clinical features and/or survival have been addressed.

ASC expression and methylation status in lung cancer [i.e., small cell lung cancer (SCLC) and non-small cell lung cancer (NSCLC)], were evaluated in three studies (Virmani et al., 2003; Machida et al., 2006; Zhang et al., 2006). In non-malignant tissue, ASC was expressed, whereas reduced ASC expression was found in 75% of primary lung carcinoma (Machida et al., 2006) with aberrant methylation among 27–47% of tumor samples, predominantly in NSCLC (Virmani et al., 2003; Machida et al., 2006; Zhang et al., 2006). Virmani et al. (2003) did not find any correlation between the methylation status and a specific NSCLC histologic subtype, tumor stage, or lymph-node metastases, whereas in the other two studies (Machida et al., 2006; Zhang et al., 2006), methylation was identified as an independent unfavorable prognostic factor in multivariate analysis (Zhang et al., 2006), and correlated with lymphatic invasion, lymph-node metastases, and advanced disease (Machida et al., 2006), respectively. Patients with unmethylated tumors had increased survival (Zhang et al., 2006), and ASC methylation in sputum DNA predicted prognosis in patients resected for early stage disease (Machida et al., 2006). Collectively, it is agreed that in lung cancer, ASC aberrant methylation associates with negative clinical outcome.

Aberrant methylation was found in 31% of neuroblastomas (Alaminos et al., 2004). A higher frequency of methylated tumors were found at more advanced-stage disease, and a higher frequency of unmethylated tumors were found in patients with spontaneous remission. Patients with methylated tumors also had a shorter survival compared with those with unmethylated tumors (Alaminos et al., 2004), suggesting a clinically relevant pro-apoptotic role for ASC in neuroblastoma.

Two studies (Kato et al., 2008; Wu L. et al., 2016) evaluated ASC expression and methylation status in gastric cancer, with very similar findings. The methylation rate in tumors was 32–34%, and ASC expression was reduced in tumors compared to normal gastric tissue. The methylation status in the tumor correlated with the primary tumor size and lymph node metastases in multivariate analyses (Wu L. et al., 2016); patients with methylated tumor exhibited shorter survival (Kato et al., 2008; Wu L. et al., 2016). Collectively, reduced ASC expression in gastric cancer seems to correlate with worse clinical outcome.

In two studies (Akahira et al., 2004; Terasawa et al., 2004), ASC aberrant methylation was found in 19–40% of ovarian cancers, respectively. Terasawa et al. (2004) reported an association between ASC methylation and clear cell-type tumors, whereas they did not find any correlation with prognosis. Collectively, alteration in ASC expression does not seem to have a clear clinical impact in ovarian cancer.

High frequency of hypermethylation (63–65%) was found in primary prostate cancer (Collard et al., 2006; Das et al., 2006). Of note, high frequency of methylation (35%) was also detected in benign prostate hyperplasia (Das et al., 2006) and in 64% of high-grade intraepithelial neoplasia (Collard et al., 2006). As a clinical correlate, a high rate of ASC methylation in non-tumor adjacent tissue was found in patients with biochemical recurrence. However, no correlation with survival was observed (Collard et al., 2006), not supporting clinical relevance for ASC expression in prostate cancer.

In pancreatic cancer ASC expression in the tumor, evaluated by immunohistochemistry, was upregulated in over 90% of tumor samples compared with surrounding tissue (Brunetto et al., 2019). Analysis in ASC expression distribution within the tumor showed that, in addition to the expression in tumor cells, ASC was also highly expressed in TAMs in all samples. Patients with ASC mRNA expression inferior to the median value had a significantly increased survival (Brunetto et al., 2019), suggesting that ASC expression in pancreatic cancer correlates with an unfavorable clinical outcome.

Lastly, conflicting results were obtained in glioblastoma and oral squamous cell carcinoma. Aberrant methylation was present in 43% of glioblastomas, whereas ASC was unmethylated and expressed in normal brains (Stone et al., 2004). No correlation was found with age, gender or treatment, although a trend in increased survival for patients with unmethylated tumors was observed (Stone et al., 2004). In another study (Martinez et al., 2007), hypermethylation was present in 21% of glioblastomas and, at variance with the previous report (Stone et al., 2004), methylation was significantly more frequent in long-term survivors. In agreement with this finding, a tendency for better outcome in patients with methylated ASC was also observed (Martinez et al., 2007). Collectively, the two studies yielded opposing results in terms of clinical relevance, possibly because of the limited number of cases studied. Further investigations are needed to better define the contribution of ASC expression in glioblastoma.

Concerning oral squamous cell carcinoma, one study (Shimane et al., 2013) found ASC highly expressed in normal oral mucosa, whereas expression was reduced in tumors as a function of the differentiation grade (well, moderately and poorly differentiated). ASC expression correlated with tumor site, T-classification, clinical stage, mode of invasion, histopathological differentiation, and lymphocytic infiltration. Survival rate was significantly higher in patients with a higher score (increasing with the percentage of positive cells) (Shimane et al., 2013). On the contrary, in another study (Wu C.S. et al., 2016), ASC expression was higher in the tumor compared to the adjacent normal mucosa, and its expression correlated with tumor stage, node involvement, extracellular spread, perineural invasion, and tumor depth. Patients with higher ASC expression showed reduced survival and in multivariate analysis ASC upregulation was an independent predictor of survival (Wu C.S. et al., 2016). Possible explanations for these contradictory results might be the different scoring methods for immunohistochemistry. Due to this important discrepancy in survival, the clinical relevance of ASC expression in oral squamous cell carcinoma cannot be definitively ruled out.

## Concluding Remarks

Through diverse activation stimuli and not completely understood receptor-ligand interactions, ASC can start two different intracellular signaling pathways (i.e., apoptosis and inflammasome maturation) (Figure 1).

In tumors, ASC can be either downregulated, mostly by aberrant methylation, or upregulated in tumor cells and overexpressed in the myeloid compartment (mostly TAMs) within the tumor microenvironment.

ASC can be expressed in normal epithelial cells or non-tumor adjacent tissues, and when it is downregulated in tumor cells, the prevalent role is usually anti-tumor by activation of apoptotic pathways. On the contrary, when tumor cells have upregulated ASC expression compared with that of the normal tissue counterpart, ASC is more commonly associated with tumor-promoting functions, either by inducing the release of pro-inflammatory cytokines, or by acting as danger signals when released in the extracellular space as specks, thus perpetuating inflammation. Indeed, ASC expression in myeloid cells is associated with tumor-promoting inflammation.

ASC can be targeted by therapeutic strategies, which are already in the clinical practice or under development, aimed to either increase its expression (i.e., demethylating agents) or interfere with inflammasome components (Coll et al., 2015). However, due to the opposite anti-tumor or pro-tumor ASC functions, future therapeutic applications for ASC targeting should well monitor tissue- and cell type-specific tumor contexts.

## Author Contributions

MP and LM wrote the manuscript.

## Conflict of Interest

The authors declare that the research was conducted in the absence of any commercial or financial relationships that could be construed as a potential conflict of interest.

## Abbreviations

ASC: Apoptosis-associated Speck-like protein containing a CARD
CAF: cancer associated fibroblast
CARD: caspase activation and recruitment domain
IL: interleukin
NSCLC: non-small cell lung cancer
TAMs: tumor associated macrophages
SCLC: small cell lung cancer
TMS1: Target of Methylation-induced Silencing-1
TSLP: thymic stromal lymphopoietin
